# Transcriptome Profiling and Genetic Study Reveal Amplified Carboxylesterase Genes Implicated in Temephos Resistance, in the Asian Tiger Mosquito *Aedes albopictus*

**DOI:** 10.1371/journal.pntd.0003771

**Published:** 2015-05-22

**Authors:** Linda Grigoraki, Jacques Lagnel, Ilias Kioulos, Anastasia Kampouraki, Evangelia Morou, Pierrick Labbé, Mylene Weill, John Vontas

**Affiliations:** 1 Department of Biology, University of Crete, Heraklion, Greece; 2 Institute of Molecular Biology and Biotechnology, Foundation for Research and Technology-Hellas, Heraklion, Greece; 3 Hellenic Centre for Marine Research, Heraklion-Crete, Greece; 4 Department of Crop Science, Pesticide Science Lab, Agricultural University of Athens, Athens, Greece; 5 Institut des Sciences de l’Évolution, CNRS, IRD, Université de Montpellier, Montpellier, France; Liverpool School of Tropical Medicine, UNITED KINGDOM

## Abstract

**Background:**

The control of *Aedes albopictus*, a major vector for viral diseases, such as dengue fever and chikungunya, has been largely reliant on the use of the larvicide temephos for many decades. This insecticide remains a primary control tool for several countries and it is a potential reliable reserve, for emergency epidemics or new invasion cases, in regions such as Europe which have banned its use. Resistance to temephos has been detected in some regions, but the mechanism responsible for the trait has not been investigated.

**Principal findings:**

Temephos resistance was identified in an *Aedes albopictus* population isolated from Greece, and subsequently selected in the laboratory for a few generations. Biochemical assays suggested the association of elevated carboxylesterases (CCE), but not target site resistance (altered AChE), with this phenotype. Illumina transcriptomic analysis revealed the up-regulation of three transcripts encoding CCE genes in the temephos resistant strain. *CCEae3a* and *CCEae6a* showed the most striking up-regulation (27- and 12-folds respectively, compared to the reference susceptible strain); these genes have been previously shown to be involved in temephos resistance also in *Ae*. *aegypti*. Gene amplification was associated with elevated transcription levels of both *CCEae6a* and *CCEae3a* genes. Genetic crosses confirmed the genetic link between *CCEae6a* and *CCEae3a* amplification and temephos resistance, by demonstrating a strong association between survival to temephos exposure and gene copy numbers in the F2 generation. Other transcripts, encoding cytochrome P450s, UDP-glycosyltransferases (UGTs), cuticle and lipid biosynthesis proteins, were upregulated in resistant mosquitoes, indicating that the co-evolution of multiple mechanisms might contribute to resistance.

**Significance:**

The identification of specific genes associated with insecticide resistance in *Ae*. *albopictus* for the first time is an important pre-requirement for insecticide resistance management. The genomic resources that were produced will be useful to the community, to study relevant aspects of *Ae*. *albopictus* biology.

## Author Summary

Some of the most immediate challenges that the world faces are caused by insecticide-resistant mosquitoes that seriously threaten human health, via the diseases they transmit. Temephos is a major larvicide that has been used extensively for the control of *Ae*. *albopictus* and its often sympatric *Ae*. *aegypti*. Here we identified temephos resistance, and showed that specific carboxylesterase genes are overexpressed in the resistant strain through gene amplification. It is striking that exactly the same CCE genes, namely *CCEae6a* and *CCEae3a*, which are clustered in *Ae*. *aegypti* genome, have also been found associated with temephos resistance in this species. Identification of genes responsible for insecticide resistance is a key step in order to make careful risk assessments regarding the emergence of resistance and to design effective and sustainable vector control strategies. The gDNA—resistance associated marker (i.e.: the gene amplification which was confirmed to be genetically linked with the phenotype) can be used to follow the dynamics of resistance in the field, as well as facilitate population genetic studies for this highly invasive vector. The transcriptomic data that were produced represent a significant genomic resource, which will facilitate molecular studies in *Ae*. *albopictus*.

## Introduction

The Asian Tiger mosquito *Ae*. *albopictus* is a major vector for a variety of viral diseases, such as dengue fever and chikungunya, which threaten over 2.5 billion people worldwide. Trade and climate changes have opened new ecological niches to this highly invasive species in temperate areas of the world. In Europe it was first detected in Albania in 1979[1] and since then it has spread to all Mediterranean/S. European countries including Greece, as well as Germany, Switzerland and The Netherlands[2]. Its invasive success has been associated with its ability to survive under cooler temperatures, compared to other mosquito species[3,4]. Cases of epidemics of viral transmission (chikungunya) that recently appeared in Europe and elsewhere (La Reunion Island in 2005 and 2006; Italy 2007, France and Croatia 2010, Portugal 2012) were directly associated with the expansion of *Ae*. *Albopictus* [5]. *Ae*. *albopictus* is also a severe nuisance for humans, as it is an extremely aggressive exophilic feeder, biting throughout the day.

The control of *Ae*. *albopictus* relies on clean-up campaigns that reduce the larval breeding sites, repellents (spatial or personal), and insecticides (both larvicides and adulticides). Temephos is an organophosphate (OP) larvicide which has been used for many decades to control *Ae*. *albopictus* mosquitoes, and the often sympatric *Ae*. *aegypti*, in several geographical regions, such as Asia and S America [6]. However, resistance against temephos has been selected and it already compromises the efficiency of the control intervention against *Ae*. *aegypti* [6,7], two regions that harbor the greatest burden of viral diseases transmitted by *Aedes* vectors. The levels of temephos resistance in *Ae*. *albopictus* seem to be relatively low at present, however there are indications that the trait is evolving[7]. As only a limited number of larvicides are available on the market, temephos resistance is an important issue for several countries where it remains a main active ingredient. It is also a concern for regions that have banned its use, such as Europe: this molecule is a potential reliable reserve, for emergency epidemics or new invasion cases.

Understanding insecticide resistance mechanisms is an important pre-requirement for the subsequent development of tools and practices that can improve the management and sustainability of control programs. There are two main molecular mechanisms responsible for insecticide resistance: target site resistance, due to mutations that reduce the binding affinity of the insecticide with its molecular target, and metabolism-based resistance, due to changes in detoxification enzymes (such as cytochrome P450s, carboxyesterases (CCEs), Glutathione S-transferases (GSTs), ABC transporters, and UGD-transferases (UGTs)), which sequester, metabolise or facilitate the secretion of the insecticide molecules, thus preventing them from reaching their target [8–10]. The G119S substitution in acetylcholinesterase-1 (AChE1) has been documented to confer resistance against OPs and carbamates (CARB) insecticides in *Culex pipiens* and *Anopheles gambiae* mosquitoes [11,12]. Metabolic resistance to OPs has also been associated with the over-production of CCEs, such as the Est-2 and Est-3 in *Culex pipiens* complex and the *CCEae3a* and *CCEae6a*, more recently, in *Ae*. *aegypti* [13].Gene amplification and transcriptional up-regulation, alone or in combination, seem to be responsible for the increased production of esterases in insecticide resistant mosquitoes [14,15].

Here, we report the use of classical approaches, combined with transcriptomics to investigate the molecular mechanisms of temephos resistance identified in an *Ae*. *albopictus* population isolated from S. Europe. Significant genomic resources have been developed, and genes strongly implicated in *Ae*. *albopictus* temephos resistance have been identified.

## Materials and Methods

### Mosquito strains and toxicity bioassays

Three different *Ae*. *albopictus* strains were used in this study: Lab, a reference susceptible strain, which was originally collected in Malaysia[16] was very kindly provided by Dr Charles Wondjii (Liverpool School Tropical Medicine, UK); Par-GR, a strain derived from an *Ae*. *albopictus* population collected in Athens (Greece) in 2010 using ovitraps (dark plastic cup with a piece of wooden stick over the inner part of the cup and filled with tap water) placed in putative oviposition sites of *Ae*. *albopictus*; Tem-GR, a strain that was derived from the Par-GR strain by temephos selection using standard WHO larval bioassays [17] and at least 1000 larvae in each generation for 12 generations at a dose killing 80% (LC_80_) of the insects. Mosquitoes were reared in standard insectaries conditions (temperature: 27°C; relative humidity: 80%; photoperiod: 12 hours day/night). Standard WHO larval bioassays on late 3rd /early 4th instars larvae were conducted to detect the level of susceptibility to temephos [17]. At least three replicates of 20 larvae were used for each concentration. Mortality was recorded after 24 hours. To determine the LC_50_s and confidence intervals, data were analyzed using the Polo plus 2002–2014 LeOra software and the R script BioRssay v. 6.1[18].

### Enzyme activity measurements

Enzyme activity measurements were carried out in 96-well plates (NuncMaxiSorp) using a Spectra Max M2e multimode microplate reader (Molecular Devices, Berkshire, UK), following mosquito-specific assay protocols [19,20], with slight modifications. Briefly, for the carboxylesterase activity assay, individual larvae of each strain were homogenized in 600μl of 0.1M sodium phosphate buffer pH 7 containing 1% Triton X-100. 2μl from this homogenate were transferred in triplicates to a 96-well microplate and 200μl of 0.3mM α- or β- naphthyl acetate diluted in 0.02M sodium phosphate buffer pH 7.4 were added to each well. After 20min incubation, 50μl of 6.4mM Fast Blue B salt (Sigma) diluted in 35mM sodium phosphate buffer pH 7 containing 3.5%SDS were added to each well. Absorbance was measured at 570nm after five minutes of incubation.

For AChE1 activity and inhibition assays, individual larvae were homogenized in 100μl extraction buffer (0.1Μ sodium phosphate buffer pH 7 containing 1% Triton X-100) and the supernatant obtained was used as the enzyme source. The reaction was conducted in 205μl substrate–reagent solution of 0.1M sodium phosphate buffer pH7 containing 25μl enzyme source, 5,5′-Dithiobis(2-Nitrobenzoic Acid) (DTNB) and acetylthiocholine (ATCHI), in final concentration of 0.5 mM and 1.2 mM, respectively, in the presence of different concentrations (5 to 30 μΜ) of the analytical-grade inhibitors propoxur and paraoxon. AChE1 activity and residual activity (percentage inhibition) was measured and determined after 25min incubation time at 405nm. The protein concentration in the enzyme source for all biochemical assays was determined according to Bradford (1976), using bovine serum albumin as a standard, to normalize activities for protein concentration. At least three biological replicates for each strain of at least 20 larvae were tested. The mean activity values were compared between the resistant and the susceptible strains, by Mann-Whitney test and differences were considered significant at a *p*<0.05.

### Extraction of gDNA and RNA, cDNA synthesis, and preparation and validation of Illumina libraries

Several batches of five to ten larvae from each strain were used for gDNA extraction using the DNeasy Blood and Tissue Kit (Qiagen) and the Cethyl Trimethyl Ammonium Bromide extraction method, as described in [21]. The resulting DNA was resolved in 100μl or 20μl water, respectively and samples were treated with RNase A (Qiagen) to remove RNA. Several batches of five to ten larvae in late third to early fourth stage were used for RNA extraction respectively, using the Arcturus Picopure RNA Extraction Kit (Arcturus, California, USA). RNA was treated with DNAse I (RNase–Free DNase Set Qiagen) to remove genomic DNA contamination and subsequently used for cDNA synthesis, either using Superscript III reverse transcriptase and Oligo-dT 20 primers (Invitrogen) (qPCR), or using Mint-Universal cDNA Synthesis kit. For Illumina sequencing, libraries were prepared in accordance with the Illumina Tru Seq RNA sample preparation guide (May 2012, rev. C) for Illumina Paired-End Indexed Sequencing http://www.biotech.wisc.edu/Libraries/GEC_documents/TruSeq_RNA_SamplePrep_v2_Guide_15026495_C.pdf. Briefly, poly-A mRNA were first purified using Illumina poly-T oligo-attached magnetic beads and two rounds of purification. During the second elution of the poly-A-RNA, the mRNA was also fragmented and primed with random hexamers for cDNA synthesis. Cleaved mRNAs were reverse transcribed into first strand cDNA using reverse transcriptase and random primers. The RNA template was then removed and a replacement strand synthesized to generate double-stranded cDNA. Ends were subsequently repaired, dA base added, and Illumina indexing adapters were ligated. Finally, cDNA fragments that have adapter molecules on both ends underwent 15 cycles of PCR to amplify the amount of prepared material. The resulting libraries were validated using the Agilent 2100 bioanalyser to confirm the concentrations and size distribution. Samples were quantified using a Qubit 2.0 Fluorometer, before normalizing the concentration and pooling the samples, prior to validating the pool to be sequenced using qPCR. The pool was loaded at a concentration of 8pM onto 1 lane of an Illumina flow cell v3. The sample was then sequenced using the Illumina HiSeq2000, 100bp paired end run. Two libraries (two biological replicates) derived from independent RNA preparations from each strain, and were sequenced twice (two technical replicates) using Illumina platform, with sequenced paired end reads size equal to 100 bases.

### Analysis of transcriptome profiling data

#### Read pre-processing

Read quality and pre-processing (adaptor removal, quality and size trimming, low complexity filtering and rRNA removal) were performed using Scythe (https://github.com/vsbuffalo/scythe) and SeqPrep (https://github.com/jstjohn/SeqPrep) for adaptor removal. Filtering and reads trimming (Q:25, read size>49) was performed with sickle (Joshi NA, Fass JN. 2011. https://github.com/najoshi/sickle) and low complexity sequences trimming was performed using PrinSeq [22]. To estimate ribosomal and mitochondrial RNA, the NCBI rRNA dataset from genus *Aedes*, the SILVA rRNA database version 111 (http://www.arb-silva.de) as well as the NCBI *Ae*. *albopictus* mitochondrial genome were used. Due to rRNA contamination heterogeneity between libraries (1.27%<rRNA<10.45%) the contaminant rRNAs were removed.

#### Assembly

The de novo assembly was performed on the pre-processed (singleton and re-paired) reads of combined libraries using Trinity package [23] Release: 2013-11-10 with default parameters and the minimum contig size fixed to 200bp). The quality of the assembled data was assessed following four procedures: (a) align back the reads to the assembly with Bowtie2 [24], (b) examine the similarity between the Trinity assembly and nr NCBI protein database (c) estimate the completeness of the product predicting the longest open reading frames (ORF) of the contigs using a custom perl script based on the EMBOSS program followed by a blastp (E-value< 1e-20) using the peptide datasets of the ENSEMBL v24 mosquitos and 21 NCBI full CDS of *Ae*. *aegypti* and (d) determine how many of 248 highly conserved eukaryotic genes were present in the *Ae*. *albopictus* transcriptome with the program CEGMA [25]. No filtering of low-abundance contigs was performed in order to maximize the detection of low expressed transcripts.

#### Similarity searches and detection of sequences related to the insecticide target and detoxification

For sequence annotation of the assembled transcripts, a blastx similarity search against the NCBI protein database *nr* (*e*-value threshold 10^–6^; keeping the top 20 hits) was performed using the parallel version of NOBLast [26]. Based on the blastx results (first best Hit, E-value <1e-10), genes of interest (ie detoxification genes, and genes encoding putative insecticide targets) were searched by a perl/SQL scripts using regular expression. Putative orthlogues were “named” based on their best blast hit with *Ae*. *aegypti*. The results were manually checked.

#### Differential gene expression analysis

Differential expression (DE) analysis was performed at the “gene” (component) level by pair wise comparisons between the parental and the selected strains (using the 2 biological replicates). The DE analysis was performed for each technical replicate (lane) separately. Only the differential expressed genes commonly up- or down-regulated between the two 2 technical replicates were retained. For the quantification, the paired reads of each sample were aligned to the transcriptome assembly with Bowtie2 and abundance was estimated with RSEM v-1.2.4, as implemented in the trinity script *run_RSEM_align_n_estimate*.*pl*. The estimated expected counts for each sample (the sum of counts in each row >10) were extracted and used for the analysis of differential expression conducted in EdgeR-3.8.0 bioconductor package [27] using EdgeR-robust method to dampen the effect of outliers genes [28] with logFC>1, FDR<0.05 and CPM>1 in at least 2 samples. The computations were performed at the HCMR-Crete, high-performance computing bioinformatics platform.

### Quantitative real time PCR

The levels of selected transcripts were measured by quantitative PCR (qPCR). Amplification reactions of 25μl final volume were performed on a MiniOpticon Two-Color Real-Time PCR Detection System (BioRad) using 2μl of cDNA (diluted 25 times),0.2μM primers (S1 Table) and Kapa SYBR FAST qPCR Master Mix (Kapa-Biosystems). Two housekeeping genes histone 3 and the ribosomal protein L34 were used as reference genes for normalization [29]. A fivefold dilution series of pooled cDNA was used to assess the efficiency of the qPCR reaction for each gene specific primer pair. A no template control (NTC) was included to detect possible contamination and a melting curve analysis was done in order to check the presence of a unique PCR product. Experiments were performed using four biological replicates and two technical replicates for each reaction. Relative expression analysis was done according to Pfaffl [30] and significance of calculated differences in gene expression was identified by a pair-wise fixed reallocation randomization test. Quantitative PCR reactions for the gDNA analysis were performed as described above, using gDNA as template. Histone 3 was used as a reference gene for the analysis of the gDNA [30].

### Crosses and genetic association of esterase gene amplification with resistance

Approximately fifty resistant females (Tem-R) were crossed to fifty susceptible (Lab) males (Fem Res x Male Sus) and fifty susceptible females to fifty resistant males (Fem Sus x Male Res), in two replicates. Susceptibility to temephos of the F1 generation from both crosses was determined with a bioassay, as described above and dose-response curves were produced as described in the BioRssay manual [18]. Late third to early fourth instar larvae (F1 generation) of the Fem Res x Male Sus cross were selected with 0.05ppm temephos, a concentration which kills >90% of the susceptible individuals. This selection step was introduced to ensure that only heterozygous, but no susceptible individual would be isolated, in case the resistant strain is not completely homogenous. After that, F1 survivors were intercrossed and their eggs were collected and let to hatch. Late third to early fourth instar larvae of the F2 generation were selected with 0.12ppm temephos and larvae which died after 4 hours of exposure (approximately 60 to 80%), as well as larvae which survived after 24 hours of exposure were collected. Genomic DNA was extracted from individual larvae and used as template in a quantitative real time PCR, performed as described above, in order to compare copy numbers of particular genes between dead (the most susceptible) and surviving (the most resistant) larvae. In this case results were expressed as the reverse ratio of the esterase gene Ct over the histone 3 Ct. Ct refers to the cycle at which the fluorescence for each gene rises appreciably above the background fluorescence.

## Results

### Temephos resistance and carboxylesterase activities

The *Ae*. *albopictus* susceptible reference laboratory strain (Lab) that was obtained in order to facilitate the resistance analysis has an LC_50_ to temephos of 0.020ppm, while the most susceptible field population (Field-S-IT) from the Mediterranean region (Genoa, Italy) has an LC_50_ of 0.003 ppm (Table 1, [7]). The field resistant population collected from Greece (parental strain, Par-GR) has an LC_50_ of 0.048ppm (resistance ratio 16-fold, as compared to the Field-S-IT population). The selected resistant strain Tem-GR, obtained from selection of Par-GR with temephos, has an LC_50_ of 0.128ppm (resistance ratio 42.6-fold and 6.4-fold, as compared to the Field-S-IT and the Lab strain, respectively) (Table 1).

**Table 1:** Temephos toxicity in the temephos–selected resistant strain (Tem-GR) as compared to its parental (Par-GR), a reference laboratory strain (Lab) and a susceptible field population from Italy (Field-S-IT) [7].

Mosquitoes	LC_50_ (95% CI)	RR_50_ ^1^	RR_50_ ^2^
*Strains/field populations*			
Field-S-IT	0.003	-	1
Lab	0.020(0.018–0.024)	1.0	6.6
Par-GR	0.048(0.040–0.063)	2.4	16.0
Tem-GR	0.128 (0.106–0.160)	6.4	42.6
*Crosses*			
Female Tem-GR x Male Lab-S	0.082(0.072–0.094)	4.1	27.3
Female Lab-S x Male Tem-R	0.062(0.038–0.091)	3.1	20.6

LC_50_ values are in ppm (μgr/L).

^1^Resistant ratios calculated over the Lab-S strain

^2^Resistant ratios calculated over the Field-S-IT field population [6,7], collected in 2002 from neighboring region (Genoa, Italy).

Resistance mechanisms were investigated in the Tem-GR strain and compared to the Par-Gr and to the Lab strains. In order to determine the relative role of the most relevant carboxylesterase detoxification system, based on previous studies on temephos resistance in other species [13] versus possible alterations in the AChE target site of temephos in the resistant phenotypes, we analysed enzymatic activities of the different strains.

**Fig 1:**
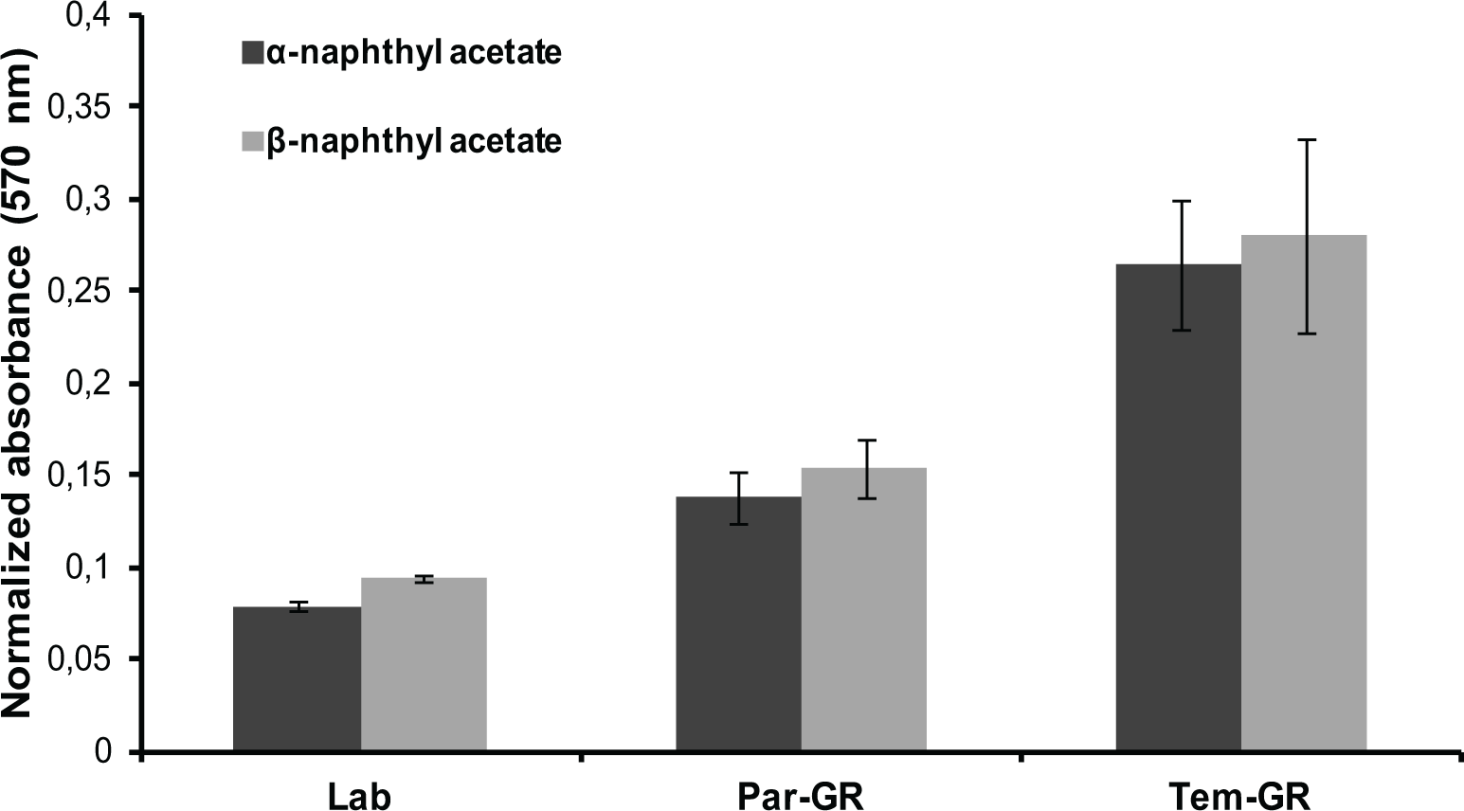
Comparison of esterase A and B activity between the resistant (Tem-GR), parental (Par-GR) and susceptible (Lab) *Aedes albopictus* strains. Absorbance measured at 570nm was normalized over the amount of protein added. Differences among all three strains for both α- and β-esterase activity were analyzed with a Mann-Whitney test and found statistically significant (p-value<0.05).

No significant differences in the AChE—ATCHI activity and/or inhibition patterns with paraoxon and propoxur, were observed among the three strains (Lab; Par-GR; Tem-GR) tested. However, α- and β-esterase activities with the substrates α- and β-naphthyl acetate were substantially different (Fig 1) among the three strains: the resistant strain Tem-GR had the highest activity followed by the Par-GR and the reference strain (Lab), in line with the temephos LC_50_ values.

### Transcriptomic analysis

In order to investigate the underlying molecular mechanisms responsible for the temephos resistance phenotype, we used a high throughout IlluminaHiSeq2000 sequencing approach. Both Par-GR and Tem-GR were reared in parallel in the lab for 12 generations. The Par-GR was used as a “susceptible” population, in order to minimise the stochastic variation, i.e. genetic background, geographical differences and impact of extended laboratory colonisation.

#### Read assembly and annotation

IlluminaHiSeq2000 sequencing of 4 cDNA libraries (herein called samples) from two *Ae*. *albopictus* strains (called conditions) yielded more than 230 Million paired-end reads for the two technical replicates (2 lanes). The pre-processing process resulted to 183.8 Million paired-reads (36.2Gbp). The cleaned short read sequences were deposited in the Sequence Read Archive (SRA) (http://www.ncbi.nlm.nih.gov/bioproject/282718; SubmissionID: SUB923821; BioProject ID: PRJNA282718).

De novo assembly performed using Trinity produced 254,336 contigs (mean length: 719.98bp, N50: 1,160bp) corresponding to 146,372 unigenes (a set of contigs which are believed to belong together).

To evaluate the accuracy of the assembled sequences (transcripts), all the usable sequencing reads were aligned onto the transcripts using Bowtie2. 86.28% of reads were successfully back aligned on this assembly and at least 79.15% of the aligned reads were properly paired.

Similarity between the Trinity assembly and nr NCBI protein database was examined using Blastx and disclosed 37,414 contigs with a coverage of > 80%. In addition similarity with a dataset of the ENSEMBL v24 mosquitoes and 21 NCBI full CDS of *Ae*. *aegypti* using blastp disclosed 11,292 contigs that meet the criteria set (min. 60% identity and min. coverage of the subject 80%). The program CEGMA identified a total of 237 (95.6%) of the 248 CEGs, and 138 of these were considered complete (75.8% of the protein identified).

The blastx search (E-value cut-off <10E-6) returned 63,978 (25.2%) contigs with at least one blast hit corresponding to 36,455 (24.9%).

### Homology searches: Transcripts encoding putative detoxification genes and targets of insecticides

Homology searches focusing on genes which encode detoxification enzymes revealed a large number of putative P450 unigenes (203 unigenes), ABC transporters (140 unigenes), esterases (113 unigenes), GSTs (41 unigenes) and UGTs (4 unigenes) (Tables 2 and S2). Several transcripts that encode putative targets of insecticides were also identified, including AChEs, the target site of OPs and CARBs, sodium channel, the target site of pyrethroids, nicotinic acetyl-choline receptors (nAChRs), target site of neonicotinoids, and chloride channels, the putative target site of avermectins (Tables 2 and S2).

**Table 2:** Annotated *Aedes albopictus* genes encoding for detoxification and/or redox genes and insecticide target subunits.

Family	Contigs	Unigenes	Average contig Length (aa)	Number of blast hits
*Detoxification (Phase I*, *Phase II and Phase III)*				
ABC transporter	230	140	344.909	54
Esterase	195	113	291.887	75
Carboxyl Cholinesterase	65	35	308.708	25
Hydrolase	124	72	286.677	54
P450 oxidases	573	203	268.408	143
Cytochrome B	13	8	179.615	8
Cytochrome b5 reductase	2	1	327	1
Glutathione S-Transferase	92	41	179.815	34
Glutathione peroxidase	8	2	237.875	3
UDP/UGTs (Glucosyl/ Glucuronosyl Transferase)	4	4	285.25	3
*Redox genes*				
Superoxide dismutase	6	5	169	6
Catalase	2	2	255	1
Peroxidase	42	22	272.452	12
NADH dehydrogenase	31	17	180.226	17
NADH oxidoreductase	38	24	223.605	19
*Insecticide targets*				
Chitin synthase	14	9	276.214	5
Chloride Channel	41	22	231.317	19
ACCase	10	5	314.4	3
AChE	20	12	212.25	7
GABA	20	17	165	10
Rayanodine receptor	10	8	555.6	1
Sodium Channel	32	25	179.125	9
nAChR	15	10	261.133	8

### Genes differentially expressed in insecticide resistant strain

Differential transcription analysis between the resistant and the parental strains was performed on the 146,372 unigenes that were identified by the *de novo* assembly of the Illumina transcriptome.

A total of 1,659 unigenes (S3 Table) were considered as differentially transcribed in the Tem-GR resistant strain, compared to the Par-GR parental strain, using a >4-fold regulation biological relevant threshold either direction and FDR < 0.05. Out of 1,042 transcripts which were upregulated in the resistant strain, 309 transcripts had a match with known proteins. Among them, 17 unigenes that encode putative detoxification enzymes were identified (Table 3). Genes were named based on best blast hits with *Ae*. *aegypti*. Three of them encode CCEs, the *CCEae3a* (in *Ae*. *aegypti* AAEL005112) (6.6-folds), the *CCEae6a* (in *Ae*. *aegypti* AAEL005122) (6.1-folds) and the AAEL015578 (5.6-folds) CCE. Eight cytochrome P450s, primarily members of the CYP6 family, were also upregulated (Table 3), suggesting the involvement of monooxygenase metabolic pathways in temephos resistance. The UDP-glycosyltransferases (UGTs) AAEL003079 and the AAEL001533, showed the most striking up-regulation (16.7 and 13.2-folds, respectively), among all detoxification genes, while the UGTs AAEL003076, AAEL001822, AAEL014371 also showed remarkable overexpression (Table 3). Other up-regulated transcripts that do not belong to detoxification gene families, but have been associated with insecticide resistance in other mosquito species, include transcripts with similarity to cuticle proteins (AAEL002441, CPIJ003474) (S3 Table), and proteins involved in lipid biosynthesis (such as the putative fatty acid synthase genes AAEL002204 and AAEL002227). Although several detoxification genes were found up-regulated we focused on CCEs, as their involvement in the resistant phenotype was suggested also by the biochemical analysis and previous works had indicated their involvement in temephos resistance [13,31] in the closely related *Ae*. *aegypti* (divergence dates estimated as 59 ± 19 My) [32].

**Table 3:** Over-expressed transcripts in the temephos selected resistant strain compared to the parental, encoding putative detoxification genes.

Class of gene	Unigene ID	Best BLAST hitaccession number*	Best BLAST hit gene name	Fold change	FDR-value
**Esterases**					
	comp96216_c0	AAEL005122	*CCEae6a*	6.6	1.57E-006
	comp84380_c0	AAEL005112	*CCEae3a*	6.1	4.97E-006
	comp98671_c1	AAEL015578		5.6	6.55E-005
**P450s**					
	comp84673_c0	AAF97938	*CYP6N3v3*	12.7	3.19E-010
	comp93834_c1	AAEL009127	*CYP6M11*	7.2	5.44E-007
	comp55309_c0	AAEL012144	*CYP303A1*	7.2	3.90E-004
	comp97034_c1	AAF97937	*CYP6N3v2*	6.3	7.09E-006
	comp80605_c0	AAF97945	*CYP6N4v5*	6	2.11E-003
	comp93784_c0	ADY68483	*CYP6N9*	4.6	8.20E-005
	comp97161_c0	AAEL014893	*CYP6BB2*	4.3	1.38E-003
	comp89308_c0	AEB77680	*CYP6M6*	4	1.76E-003
**GSTs**					
	comp86466_c1	AAEL001054	*GSTd4*	4.9	4.10E-004
	comp89059_c0	AAEL010500	*GSTx2*	4.1	6.92E-004
**ABC transporters**					
	comp60520_c0	AAEL012702		4	2.95E-002
**UGTs (Glucosyl/glucu-ronosyl transferase)**					
	comp84756_c0	AAEL003079		16.7	1.63E-010
	comp91317_c0	AAEL001533		13.2	1.17E-010
	comp74680_c0	AAEL003076		9.7	3.82E-008
	comp89108_c0	AAEL001822		4.9	9.03E-003
	comp71075_c0	AAEL014371		4.4	1.44E-003

Upregulated detoxification genes, identified by the differential expression transcriptomic analysis (threshold >4-fold,FDR <0.05).

*blastx against NCBI nr E value<1e-10

Out of 617 unigenes which were down-regulated in the resistant strain, 338 transcripts had a match with known proteins. Eight cytochrome P450s were present in this group (S3 Table), with the AAEL002067 and the AaCYP9J24 putative homologue showing the highest down regulation (7.8 and 6.5-fold, respectively), and the AaCYP325AA1, and AaCYPJ22 putative orthologues, showing also significant levels of down regulation (5.6 and 5.3-folds, respectively). A possible explanation for the down-regulation of P450s, in relation to organophosphate resistance might be the possible involvement of those P450s in the activation pathways of the pro-insecticide temephos, however more work is required to investigate this hypothesis.

### Gene amplification associated with elevated levels of CCE transcripts in temephos resistant strains

Quantitative PCR was used to validate the up-regulation of the carboxylesterases *CCEae3a*, *CCEae6a* and AAEL015578 in the temephos selected strain (Tem-GR).

**Fig 2:**
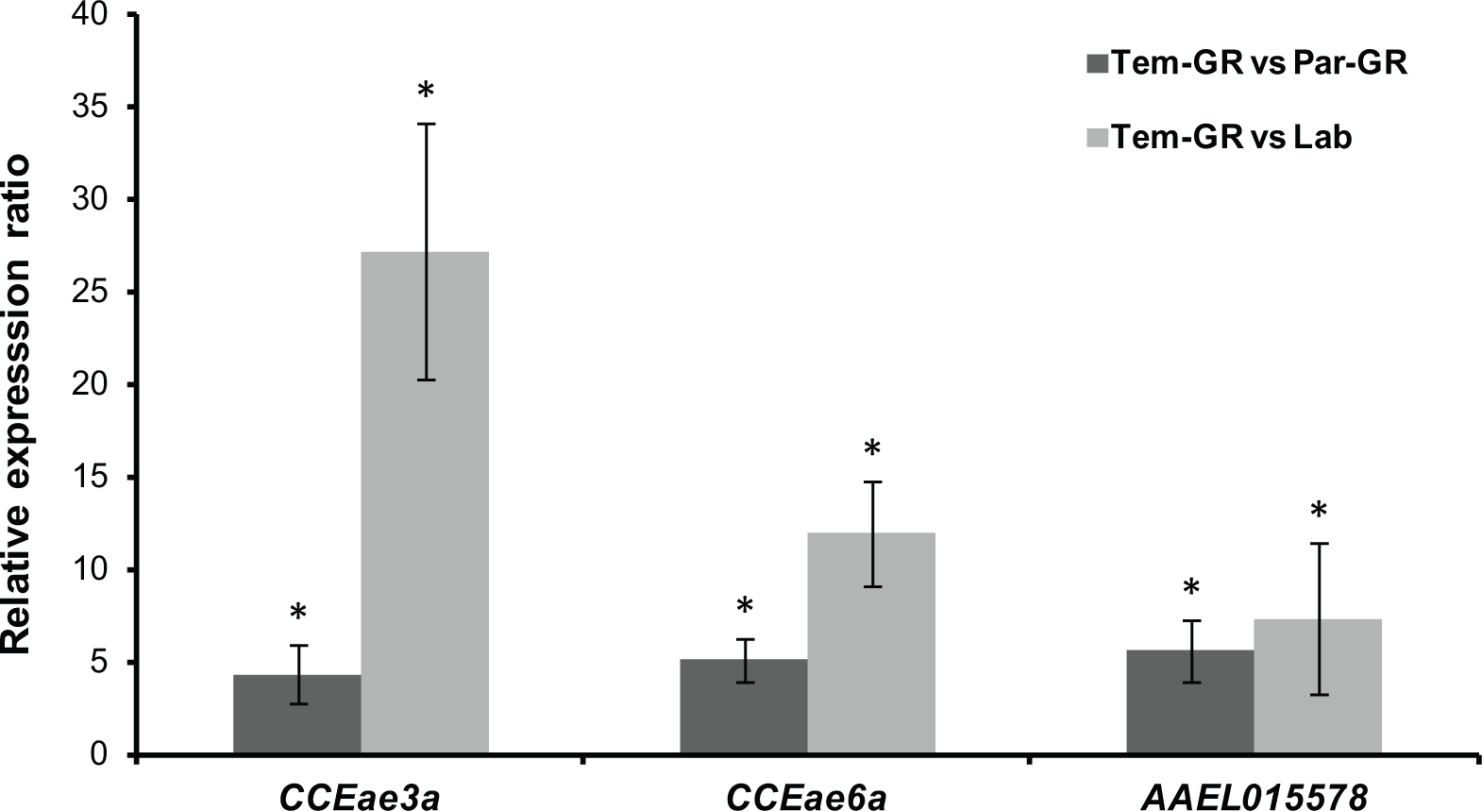
Quantification of the levels of *CCEae3a*, *CCEae6a* and AAEL015578 CCE transcripts by qPCR. Error bars represent the standard error of the calculated mean based on four biological replicates, and a star indicates statistical significance (p value<0.05)

As shown in Fig 2, the levels of *CCEae3a*, *CCEae6a* and AAEL015578were confirmed to be significantly up-regulated in the temephos resistant (Tem-GR) strain compared to the parental (Par-GR) strain, at very similar levels: the up-regulation of the *CCEae3a* was estimated at 6.1-fold by Illumina and at 4.3-fold by qPCR, the *CCEae6a*at 6.6-fold and 5-fold, and the AAEL015578 at 5.6-fold and 5.5-fold respectively. In addition, the *CCEae3a* was upregulated 27-fold in the TemR, the *CCEae6a* 12-fold and the AAEL0155787.3-fold, compared to the reference strain (Lab), respectively. This finding, is in good agreement with the bioassay and biochemical data, and indicates the involvement of the *CCEae3a*, the *CCEae6a* and the AAEL015578in the temephos resistant phenotype.

**Fig 3:**
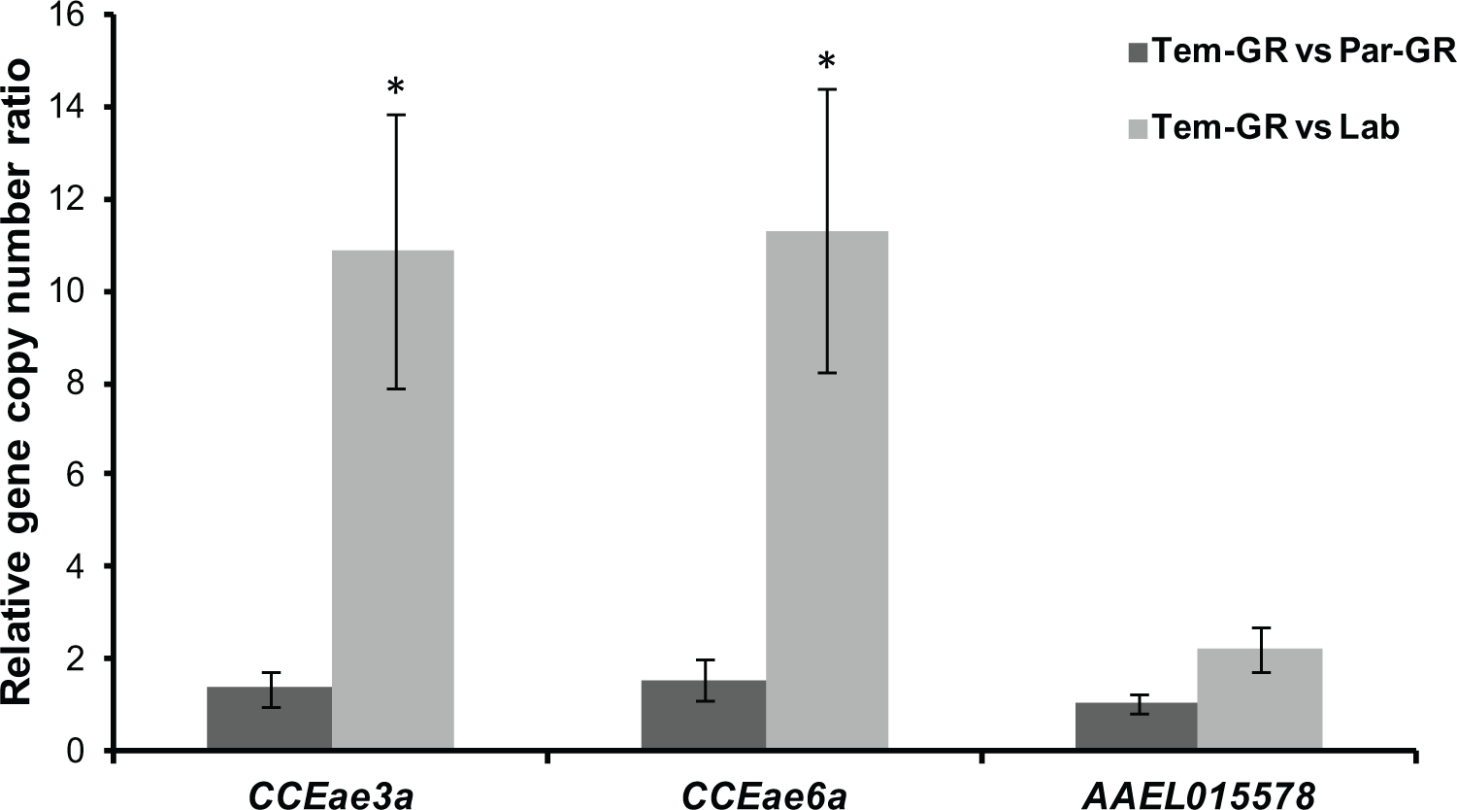
Gene copy number analysis of *CCEae3a*, *CCEae6a* and AAEL015578 CCEs. Error bars represent the standard error of the calculated mean based on three biological replicates and a star indicates statistical significance (p value<0.05).

Finally, quantitative PCR was used to compare the *CCEae3a*, the *CCEae6a* and the AAEL015578 gene copy number among the Tem-GR, the Par-GR and the Lab strains. A gene amplification of approximately 10-folds was observed for the *CCEae3a* and the *CCEae6a* in the Tem-GR, compared to the Lab strain. In contrast, copy numbers were not different for the AAEL015578 gene (Fig 3). A small and not statistically significant difference of approximately 1.8-fold was found for the *CCEae3a* and the *CCEae6a* copy numbers between the Tem-GR and the Par-GR strains, which might be due to the removal of susceptible individual mosquitoes during the selection of the heterogeneous field population.

### Resistance inheritance, and genetic links of resistance with gene amplification

**Fig 4:**
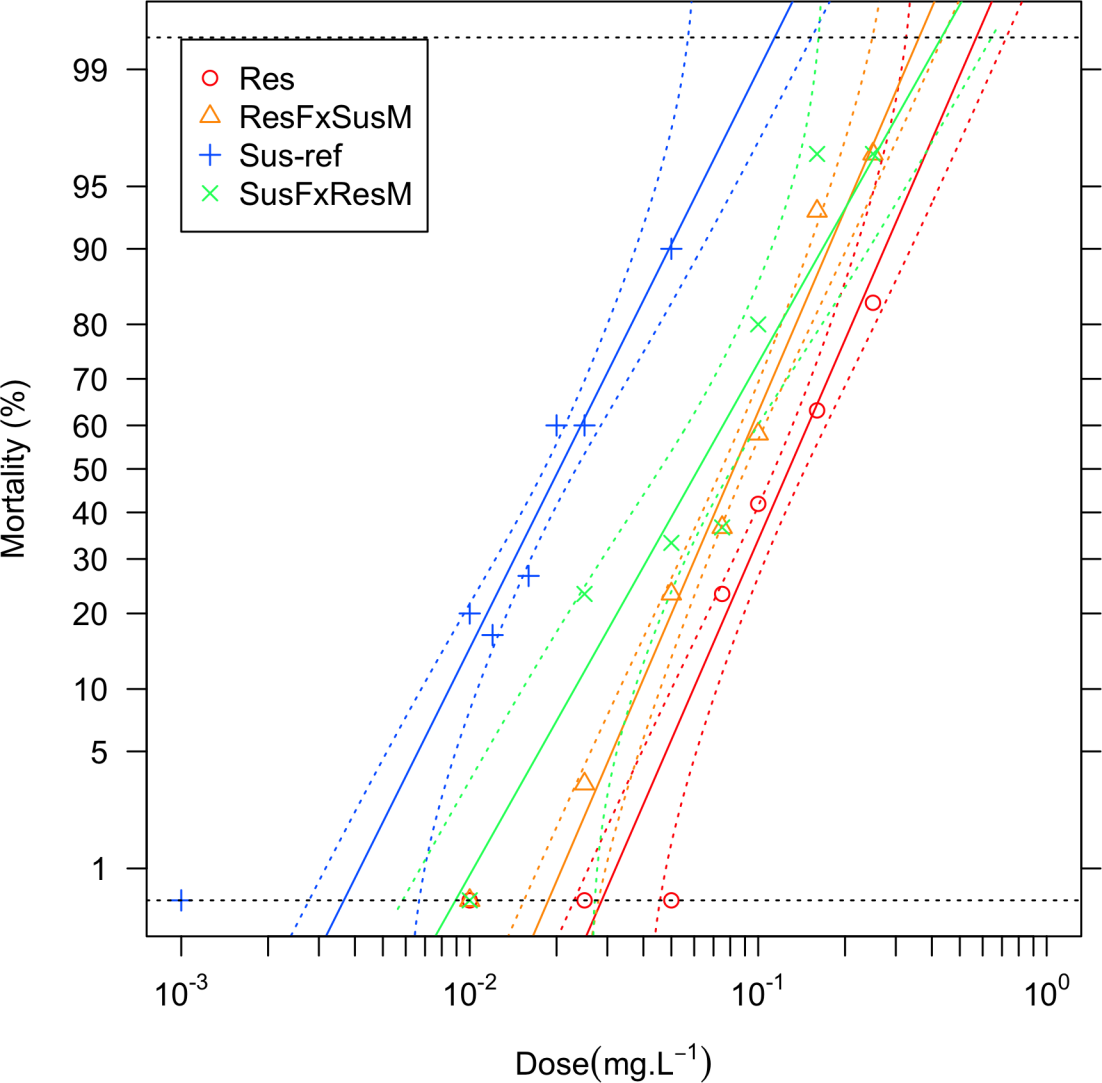
Bioassays. Bioassay results are presented as the percentage of dead larvae in relation to the temephos dose for the susceptible (Lab-S, blue crosses) and resistant (Tem-GR, red circles) strains and their F1 crosses (orange triangles and green Xs, for resistant Females x susceptible Males and for resistant Males x susceptible Females, respectively). The same-color solid lines represent the best probit fit, the dotted lines the 95% confidence interval of this fit.

Genetic crosses were performed in order to investigate the inheritance of the temephos resistant phenotype and the genetic association of the amplified *CCEae3a* and *CCEae6a* in *Ae*. *albopictus*. Resistant females (Tem-GR) were crossed to susceptible (Lab) males (Fem Res x Male Sus) and susceptible females to resistant males (Fem Sus x Male Res). F1 progeny of both crosses were tested for their susceptibility to temephos. An LC_50_ (95%CI) equal to 0.082 (0.072–0.094) was estimated for progeny of the Fem Res x Male Sus cross and an LC_50_ (95%CI) of 0.062 (0.038–0.091) for progeny of the Fem Sus x Male Res cross (Fig 4). Quantitative measurement of dominance, using Falconer’s formula [33], indicated that resistance to temephos is inherited in both cases as a co-dominant trait (D = 0.52 for Fem Res x Male Sus and D = 0.21 for the Fem Sus x Male Res, where -1 indicates complete recessive and 1 complete dominant genotype).

**Fig 5:**
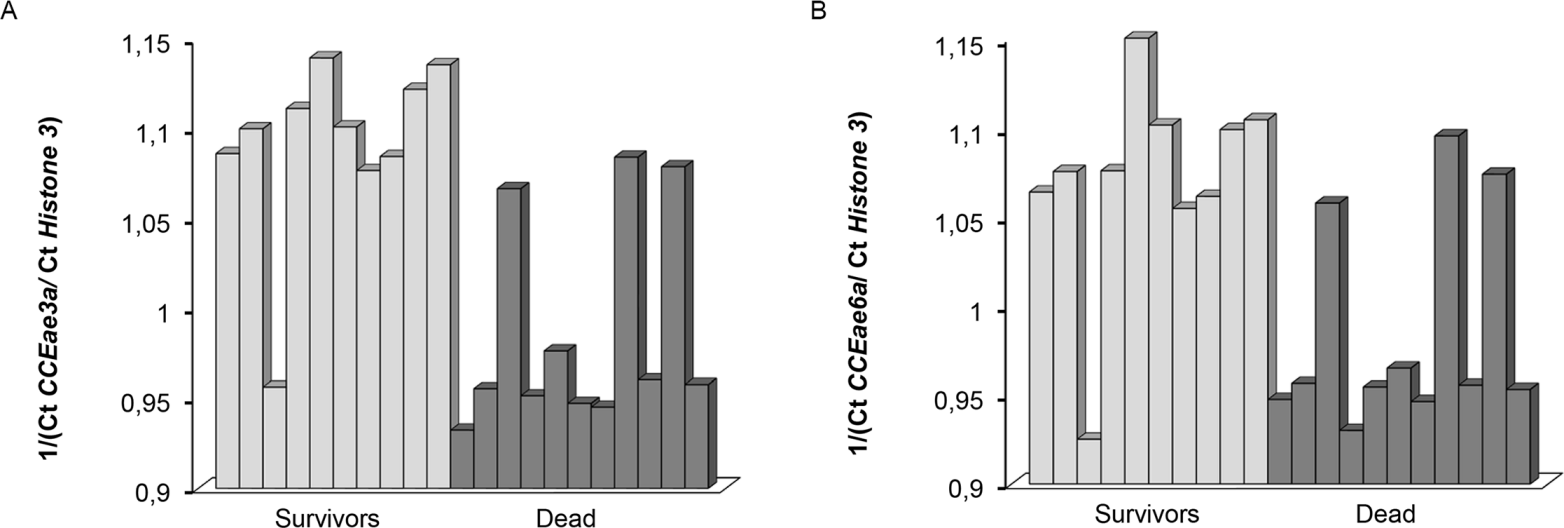
Genetic association of *CCEae3α* and *CCEae6α* copy numbers with resistance to temephos. Genomic DNA from surviving and dead F2 individuals was analyzed by qPCR. Histone 3 is used as a reference gene and values are expressed as the reverse ratio of the esterase Ct over the histone 3 Ct. A. Difference in *CCEae3α* copy numbers between survivors and dead individuals are statistically significant based on a Welsh test (p-value<0.05). B. Differences in *CCEae6α* (B) copy numbers between survivors and dead individuals are statistically significant based on a Welsh test (p-value<0.05).

Subsequently F1 individuals of the Fem Res x Male Sus cross were intercrossed and F2 progeny was obtained and selected with 0.12ppm temephos. Ten (10) dead larvae after 4h of exposure and 10 survivors after 24h of exposure were collected and quantitative real time PCR was performed using genomic DNA from individual larvae. Results showed that on average surviving larvae have statistically significant (Welsh test, p value<0.05,) more copy numbers of both *CCEae3a* and *CCEae6a* esterases compared to dead larvae (Fig 5).

## Discussion

A mosquito population of the vector of dengue fever and chikungunya virus *Ae*. *albopictus*, with reduced susceptibility to the larvicide temephos was isolated from Greece. The operational impact of resistance is not known as the study did not directly assess this issue. However, the levels of resistance observed in the field and subsequently obtained by artificial selection (16- and 42.6-folds respectively, compared to Field-S-IT) are significant. However, they may yet not substantially affect temephos performance if such resistant phenotypes are present in the field, in areas where temephos is still in use, or in cases when temephos would be required as an emergency tool (i.e. epidemics or invasion cases), given that the LC95 of the resistant population (0.6 ppm) is below the target field dose of temephos used (1 ppm), under optimum spraying conditions.

The selection of resistance to temephos could be associated with the extensive use of this larvicide in Greece over the past years [34]. However, this resistance could also have been pre-selected already in other regions, and carried along the invasive routes of the *Ae*. *albopictus* population that established in Greece.

Resistance mechanisms were subsequently investigated in a resistant strain obtained by brief laboratory selection with temephos (Tem-GR), in comparison with the colonized parental strain (Par-GR) and an “independent” reference laboratory strain (Lab). Biochemical data suggested that metabolic pathways (CCEs), but not target site resistance (altered AChE) were present and active against temephos. Illumina transcriptome analysis was used to identify genes encoding detoxification enzymes, and quantify their expression levels in the resistant strain compared to the susceptible. A large *Ae*. *albopictus* mosquito larvae transcriptome dataset, consisting of 254,336 contigs and 146,372 unigenes was produced. The full set of the raw reads have been deposited in the Sequence Read Archive (SRA) (http://www.ncbi.nlm.nih.gov/bioproject/282718; Submission ID: SUB923821; BioProject ID: PRJNA282718). The transcriptome was found to contain a number of detoxification enzymes and a number of transcripts that encode putative insecticide target subunits (Tables 2 and S2). These genomic resources will be useful to the community investigating this major vector.

Differential expression analysis revealed a number of genes with significantly differently expressed transcripts between the temephos selected (Tem-GR) strain and the parental (Par-GR), a comparison that was chosen in order to minimize stochastic variation and isolate the resistance trait in the comparison.

Three CCEs, *CCEae6a*, *CCEae3a* and AAEL015578 were among the most upregulated hits in the transcriptomic comparison between the Par-GR and the selected and more resistant Tem-GR. The up-regulation was confirmed by qPCR, and also against an independent reference strain (Lab). This finding indicates that CCEs were selected by temephos, a result that is in tight correlation with the biochemical data, and indicated the involvement of CCEs in the resistance phenotype. These three CCEs, *CCEae6a*, *CCEae3a* and AAEL015578 encode putative members of the alpha esterase clade, a group of catalytically active CCEs that has been associated with xenobiotic detoxification functions and insecticide/organophosphate resistance, via sequestration [8]. Interestingly, *CCEae6a* and *CCEae3a* (30% and 44% identity between *Ae*. *albopictus* and *Ae*. *aegypti*, respectively) were also recently found implicated in temephos resistance in *Ae*. *aegypti* from Thailand [13].

Gene amplification was subsequently found to be associated with elevated levels of *CCEae6a* and *CCEae3a*, but not AAEL015578 transcripts. Genetic crosses confirmed the link between the amplified *CCEae3a* and *CCEae6a* with temephos resistance, by demonstrating a significant association between survivorship and gene copy numbers in the F2 generation.

Gene amplification of CCEs associated with OP resistance have also been reported in other insects [35,36] and mosquito species, such as *Culex sp* [37] and more recently, *Ae*. *aegypti* where the amplification of *CCEae3a* was associated with temephos resistance [13], in line to our study.

The levels of the elevated *CCEae6a* and *CCEae3a* gene copy numbers were lower than the respective up-regulation of the transcripts in the Tem-GR resistant strain, compared to both the Lab and the Par-GR strains, indicating that additional mechanisms may also contribute to the elevated levels of the CCE transcripts. The genetic analysis in *Ae*. *albopictus* also indicated that gene regulation might have an important role in the OP resistance for some individual mosquitoes. The operation of both gene amplification, transcriptional and translational control mechanisms to regulate the expression of CCE genes involved in insecticide resistance has been previously shown [38], and it is not clear whether gene regulation or amplification (or both) is the determining factor in resistance. Furthermore, it has been shown in population studies that amplification levels vary between individuals over time through variation in organophosphate selection pressure, and that the loss or gain of gene copies, possibly through unequal sister-chromatid exchange is also a common phenomenon in mosquitoes and aphids [35,39]. The gene amplification that was identified in this study provides a gDNA marker that can be utilized to follow such dynamics of resistance alleles in the field, and investigate their origin and selection under various environmental contexts (geographic and selective pressure histories).

Based on *Ae*. *aegypti* genome, *CCEae3a* and *CCEae6a* (but not AAEL015578, which belongs to a different contig, NW_001835964.1 http://www.ncbi.nlm.nih.gov/gene/?term=AAEL015578) are clustered together within 18.214bp on the genome (contig NW_001810264.1 within a 2.3Mbp length; *CCEae3a*, from 531.089 to 541.615 and *CCEae6a* from 559.829 to 567.390 bp, http://www.ncbi.nlm.nih.gov/gene/?term=AAEL005112), which might explain the apparently equal co-amplification of those two genes (approximately 10-fold for both the *CCEae6a* and *CCEae3a*) in the *Ae*. *albopictus* resistance strain (providing that synteny is maintained between the two species). The on-going efforts for sequencing *Ae*. *albopictus* genome will help to investigate further the mechanism of the amplification, and the striking similarities between the temephos resistance mechanisms identified in *Ae*. *aegypti* and *Ae*. *albopictus*.

Finally, several genes were also found upregulated in the Tem-GR strain, such as cytochrome P450s, members of the CYP6 family that has been implicated in insecticide resistance in other mosquito species [40], and UDP-glycosyltransferases (UGTs), enzymes that are known to participate in Phase II detoxification of xenobiotics by catalyzing their conjugation with uridine diphosphate (UDP) sugars[10]. Genes of these families have also been found co-up-regulated with putative primary phase I detoxification enzymes in other resistance studies [10], including temephos resistance in *Ae*. *Aegypti* [13]. This indicates that the co-evolution of multiple mechanisms, which may act in a coordinated manner to accelerate the detoxification, may be responsible for insecticide resistance. However, the relative contribution and/or redundancies of such individual genes and pathways in the resistance phenotype have not been studied in detail as yet.

## Supporting Information

S1 Table Primers used for the amplification of CCE transcripts of *Ae*. *Albopictus*. (XLSX)

S2 Table All annotated transcripts which encode detox genes. (XLS)

S3 Table All DE genes. (XLSX)
